# High Degree of Overlap between Responses to a Virus and to the House Dust Mite Allergen in Airway Epithelial Cells

**DOI:** 10.1371/journal.pone.0087768

**Published:** 2014-02-03

**Authors:** Korneliusz Golebski, Silvia Luiten, Danielle van Egmond, Esther de Groot, Kristina Irene Lisolette Röschmann, Wytske Johanna Fokkens, Cornelis Maria van Drunen

**Affiliations:** Department of Otorhinolaryngology, Academic Medical Center, University of Amsterdam, the Netherlands; Ludwig-Maximilians-University Munich, Germany

## Abstract

**Background:**

Airway epithelium is widely considered to play an active role in immune responses through its ability to detect changes in the environment and to generate a microenvironment for immune competent cells. Therefore, besides its role as a physical barrier, epithelium affects the outcome of the immune response by the production of various pro-inflammatory mediators.

**Methods:**

We stimulated airway epithelial cells with viral double stranded RNA analogue poly(I:C) or with house dust mite in a time course of 24 hours. In order to determine cytokines production by stimulated cells, we performed multiplex enzyme linked immunosorbant assay (ELISA).

**Results:**

We demonstrate that the temporal pattern of the genes that respond to virus exposure in airway epithelium resembles to a significant degree their pattern of response to HDM. The gene expression pattern of *EGR1*, *DUSP1*, *FOSL1*, *JUN*, *MYC,* and *IL6* is rather similar after viral (poly(I:C)) and HDM exposure. However, both triggers also induce a specific response (e.g. *ATF3, FOS,* and *NFKB1*). We confirmed these data by showing that epithelial cells produce a variety of similar mediators in response to both poly(I:C) and HDM challenge (IL1-RA, IL-17, IFN-α and MIP1-α), sometimes with a quantitative difference in response (IL2-R, IL-6, IL-8, MCP-1, MIG, and HGF). Interestingly, only four mediators (IL-12, IP-10, RANTES and VEGF) where up-regulated specifically by poly(I:C) and not by HDM. Additionally, we report that pre-exposure to HDM deregulates production of cytokines and mediators in response to poly(I:C).

**Conclusions:**

Epithelial cells responses to the HDM-allergen and a virus strongly resemble both in gene expression and in protein level explaining why these two responses may affect each other.

## Introduction

**Figure 1:**
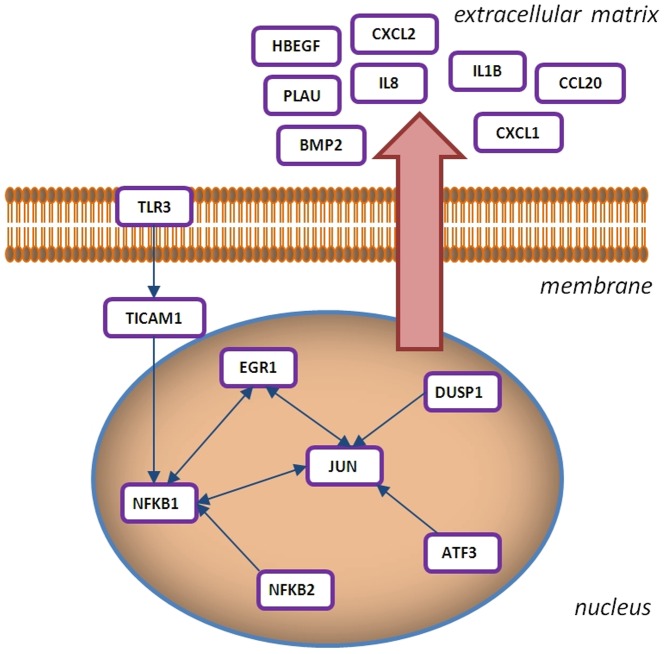
Network interaction model of the genes that make up the core response of airway epithelial cells upon HDM allergen exposure. The principal cellular locations of the gene products (extra cellular region, membrane, cytoplasm, and nucleus) are indicated and their transcriptional interaction.

Airway epithelial cells are located at the interface with the outside environment where they are able to detect and respond to changes and potential threats [1]–[5]. In this way airway epithelial cells aid the immune system by inducing recruitment of immune competent cells to local tissues and modulating their activity. Although ample data is available on responses induced by individual environmental factors in airway epithelial cells [6], potential interactions between different factors remain largely unexplored. Previously we have studied house dust mite (HDM) allergy in detail [7] and have shown that house dust mite allergen induces a pleiotrope transcriptional and proteomic response in NCI-H292 [8] and primary nasal epithelial cells [9]. We also showed that part of this response is similar in nasal and bronchial epithelial cells [10]. The shared response (or core response) of epithelium to house dust mite allergen consists of genes that have been previously linked with allergy and/or inflammation (ATF-3, EGR-1, DUSP-1, NFKB-1, etc.). Additionally, this response revealed a potential molecular link between the allergic and the viral response (Figure 1). Not only was expression of an important anti-viral receptor Toll-like Receptor 3 (TLR-3) down-regulated by exposure to house dust mite extract, but more importantly, TLR-3 triggering feeds into the transcription factor complex that is activated upon allergen exposure. Links between viruses and allergy are clinically well documented. It is clear that viral infections induce asthma/allergic exacerbations [11], [12] and viral clearance and symptoms are prolonged in allergic individuals [13]. Additionally, Rochlitzer and colleagues have recently demonstrated that chronic allergic inflammation may impair anti-viral response to acute rhinovirus infection as indicated by suppressed induction of IFN-α, IFN-γ, and IL-12 [14].

Despite reported similarities [15], studies of the possible shared molecular mechanisms behind the interplay between viruses and allergens are still missing.

To explore the response to the house dust mite allergen in airway epithelial cells in greater detail, we established a temporal expression pattern of selected pro-inflammatory transcription factors and cytokines in a bronchial epithelial cell line. We also sought to investigate whether the regulatory responses induced in airway epithelial cells by viral infections and by allergen exposure are similar and to what degree these similarities are mirrored in effector molecules production. The viral response triggered by the TLR-3 agonist poly(I:C) showed a high degree of overlap to the response to HDM provocation. Our data reveal the molecular mechanism by which viral infections and responses to an allergen may affect each other.

## Materials and Methods

### Cell culture

NCI-H292 human airway epithelial cells (American Type Culture Collection, USA) were cultured in RPMI 1640 culture medium (Invitrogen, NL) supplemented with 10% (v/v) fetal calf serum (HyClone, USA), 1.25 mM of glutamine, 100 U/mL of penicillin and 100 µg/mL of streptomycin. Cells were grown in fully humidified air containing 5% of CO_2_ at 37°C.

### Experimental set-up

House dust mite extract was kindly provided by HAL Allergy (Leiden, the Netherlands). Although the HDM extract used in this study contains traces of endotoxins, we have previously shown [16] that this biological irrelevant LPS presence does not contribute to the overall production of cytokines by the H292 cell line in our experimental setup. Polyinosinic:polycytidylic acid (poly(I:C)) was purchased from Sigma-Aldrich, NL. Cells were cultured to 80% confluence and prior to stimulation the culture medium was removed and replaced with HBSS medium (supplemented with antibiotics) for 24 hours. Cells were then stimulated with HDM (5 µg/mL) or poly(I:C) (20 µg/mL) diluted in HBSS. 5, 10, 15, 30, 60, 120, 240 minutes, 8 and 24 hours after stimulation supernatants were removed and that of 8 and 24 hours were stored at −20°C for further analysis and cells were collected for RNA extraction.

In the experiment with the HDM and poly(I:C) interplay, the H292 cell line was pre-exposed to HDM (5 µg/mL) for 24 hours, then the stimulus was removed and cells were subsequently exposed to poly(I:C) (20 µg/mL) for 8 or 24 hours. Cell free supernatants were collected and stored at −20°C for further analysis. The data shown is from one representative experiment (out of three) and each value represents an average value of three biological replicates with a standard deviation.

### RNA extraction

The total RNA from each sample was extracted by TRIzol (Life Technologies, USA) and chloroform (Merck, DE) phase separation method and additionally purified with nucleospin RNA II kit (Machery-Nagel, DE). RNA quality was checked on the Agilent 2100 bio-analyzer (Agilent Technologies, USA).

### Quantitative PCR

The concentration of RNA was determined by nanodrop ND-1000 (NanoDrop Technologies, USA). The MBI Fermentas first strand cDNA synthesis kit (Thermo Scientific, NL) was used to synthesize cDNA. Real-time PCR was performed in Bio-Rad iCycler (Bio-Rad, NL) with mRNA specific TaqMan gene expression assays (Applied Biosystems, NL) for the following genes: *ATF3* (HS00231069_M1), *EGR1* (HS00152928_M1), *DUSP1* (HS00610257_G1), *NFKB1* (HS00765730_M1), *NFKB2* (HS01028899_G1), *JUN* (HS99999141_S1), *FOS* (HS01119267_G1), *FOSL1* (HS00759776_S1), *MYC* (HS00153408_M1) according to the manufacturer’s protocol or with IQ™ SYBR Green Supermix (Bio-Rad, NL) with the following primers: *GAPDH*-forward: 5′-GAAGGTGAAGGTCGGAGTC-3′; *GAPDH*-reverse: 5′-GAAGATGGTGATGGGATTTC-3′; *β2M-*forward: 5′-TACATGTCTCGATCCCACTTAACTAT-3′; *β2M*-reverse: 5′-AGCGTACTCCAAAGATTCAGGTT-3′; *IL6*-forward: 5′-TGACAA ACAAATTCGGTACATCCT-3′; *IL6*-reverse: 5′-AGTGCCTCTTTGCTGCTTTCAC-3′; *IL1RA*-forward: 5′-CTCAGCAACACTCCTAT-3′; *IL1RA*-reverse: 5′-TCCTGGTCTGCAGGTAA-3′; *MIP1alpha*-forward: 5′-GCTGCTCAGA GACAGGAAGTCTT-3′; *MIP1alpha*-reverse: 5′- ACAGGAACTGCGGAGAGGAGT-3′. Data was analyzed in the Bio-Rad CFX Manager program (Bio-Rad, NL) and fold changes of evaluated genes were calculated using the comparative ▵Ct method. Each value was corrected for the expression of the housekeeping gene and compared to the control condition. Statistical significance (p<0.05) was determined by ANOVA and Student’s t-test using GraphPad Prism for Windows.

### Protein multiplex ELISA

Cells-free supernatants of 8 and 24 hours HDM and poly(I:C) stimulated H292 cells together with non-stimulated HBSS controls were used to determine protein levels of the following mediators: IL-1RA, IL-1β, IL-2R, IL-2, IL-4, IL-5, IL-6, IL-8, IL-10, IL-12, IL-13, IL-15, IL-17, eotaxin, TNF-α, IFN-α, IFN-γ, MCP-1, GM-CSF, G-SCF, VEGF, FGF-β, EGF, HGF, IP-10, MIG, RANTES, MIP1-α, MIP1-β, IP-10. Cytokine levels were measured with a use of a Human Cytokine Thirty-Plex Antibody Bead Kit (Invitrogen, NL) in combination with a Bio-Plex workstation (Bio-Rad, NL). All standards were diluted in HBSS medium as described by the manufacturer. Luminex software was employed for the protein concentration calculations and all concentrations are expressed in pg/mL. Fold changes were calculated by comparing the production of a cytokine in stimulated sample to non-stimulated HBSS control sample. If measured protein concentration was below the detection level, the fold change was calculated on the basis of the detection limit for the respective mediator. Statistical significance (p<0.05) was determined by ANOVA and Student’s t-test using GraphPad Prism for Windows.

## Results

### Expression profiling of the transcription factors regulated by HDM reveals different temporal patterns of expression

**Figure 2:**
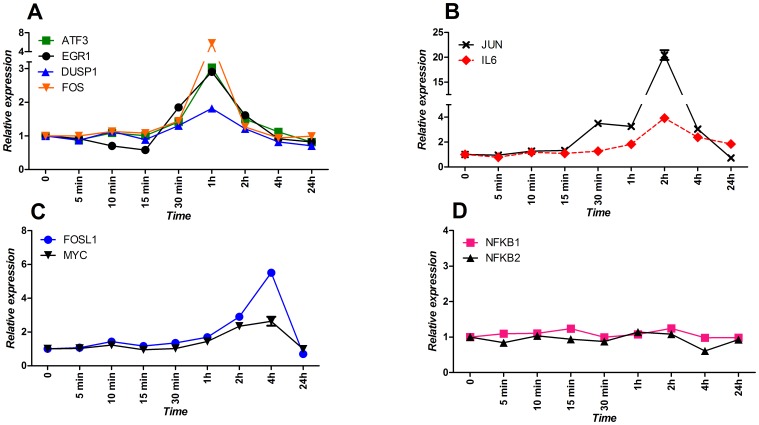
Detailed expression analysis of selected genes in response to HDM challenge. The NCI-H292 cell line was stimulated with HDM in a time course over 24 hours and expression profiles of: *ATF3*, *EGR1*, *DUSP1*, *FOS*, *FOSL1*, *MYC*, *JUN*, *IL6*, *NKB1,* and *NFKB2* were investigated. Graphs show genes that are: A) induced rapidly upon HDM stimulation; B) induced 2 hours after HDM; C) late-induced; D) not affected by HDM stimulation. Each time point represents an average of three biological replicates ± standard deviation.

Exposure of airway epithelial cells to house dust mite extract induces expression of most of the selected transcription factors. Three distinct temporal patterns emerged. Expression of *EGR1*, *ATF3*, *DUSP1*, and *FOS* (Figure 2A) are induced rapidly after stimulation and reach their maximal expression already after 60 minutes (*EGR1*: 30 min. p = 0.02, 60 min. p = 0.0003, 120 min. p = 0.003; *ATF3*: 30 min. p = 0.057, 60 min. p = 0.002; *DUSP1*: 60 min. p = 0.019.; *FOS*: 30 min. p = 0.02, 60 min. p = 0.001). The second group of transcription factors consisting of *FOSL1* and *MYC* is induced later (Figure 2C) and they reach their maximal expression only at 240 minute after induction (*FOSL1*: 120 min. p = 0.03, 240 min. p = 0.001; *MYC*: 120 min. p = 0.03, 240 min. p = 0.05). The third group takes up an intermediate position with a maximal expression level at 120 minutes and this group includes, in addition to the transcription factor *JUN*, also our positive control for induction *IL6* (*JUN*: 120 min. p = 0.001, 240 min. p = 0.02; *IL6*: 120 min. p = 0.001, 240 min. p = 0.007) (Figure 2B). Interestingly, we saw no induction of *NFKB1* and *NFKB2* (Figure 2D) that at the microarray level were affected by HDM exposure [9].

### Most transcription factors show a similar expression pattern after poly(I:C) exposure

**Figure 3:**
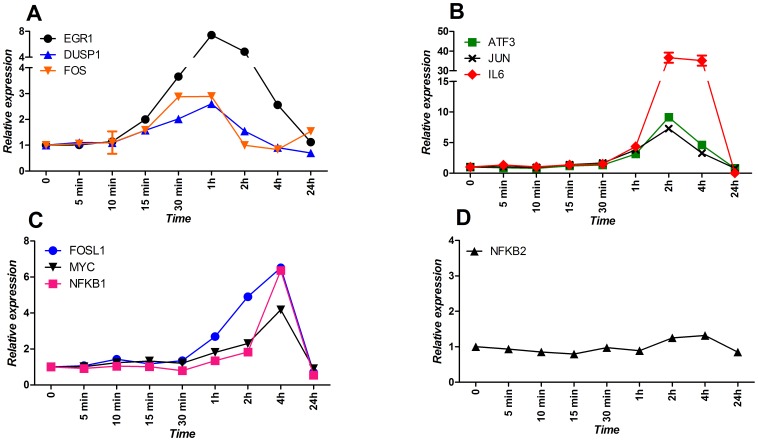
Detailed expression analysis of selected genes in response to poly(I:C) challenge. The NCI-H292 cell line was stimulated with poly(I:C) in a time course over 24 hours and expression profiles of: *ATF3*, *EGR1*, *DUSP1*, *FOS*, *FOSL1*, *MYC*, *JUN*, *IL6*, *NKB1,* and *NFKB2* were investigated. Graphs show genes that are: A) induced rapidly after stimulation with poly(I:C); B) induced 2 hours after poly(I:C); C) late-induced; D) not affected by poly(I:C) stimulation. Each time point represents an average of three biological replicates ± standard deviation.

Although the absolute level of induction varies somewhat between the different transcription factors, the profile of *EGR1*, *DUSP1*, *FOSL1, JUN*, *MYC,* and our reporter gene *IL6 (EGR1:* 30 min. p<0.0001, 60 min. p = 0.0002, 120 min. p = 0.001; *DUSP1*: 30 min. p = 0.0001, 60 min. p = 0.004, 120 min. p = 0.05; *FOSL1*: 120 min. p = 0.0002, 240 min. p = 0.04; *JUN:* 60 and 120 min. p = 0.004, 240 min. p = 0.05; *MYC*: 120 min. p = 0.0002, 240 min. p = 0.04) (Figure 3), is rather similar, with each of these genes reaching their maximal expression level at the same moment after HDM or poly(I:C) induction. However, this is not true for all transcription factors. First of all, the response of *ATF3* (60 min. p = 0.02, 120 min. p = 0.0001, 240 min p = 0.002) (Figure 3B) after poly(I:C) induction reaches its maximal level at 120 minutes when the expression after HDM exposure is on its way to return to baseline values. Secondly, and in contrast to HDM stimulation, we now see a clear up-regulation of *NFKB1* and a reduced *FOS* expression (*NFKB1*: 120 min. p = 0.02, 240 min. p = 0.001; *FOS*: for 60 and 120 min. p<0.0001) (Figure 3C). *NFKB2* is also not induced after poly(I:C) exposure (Figure 3D).

### Production and release of majority of mediators was affected by HDM and poly(I:C) challenge

**Figure 4:**
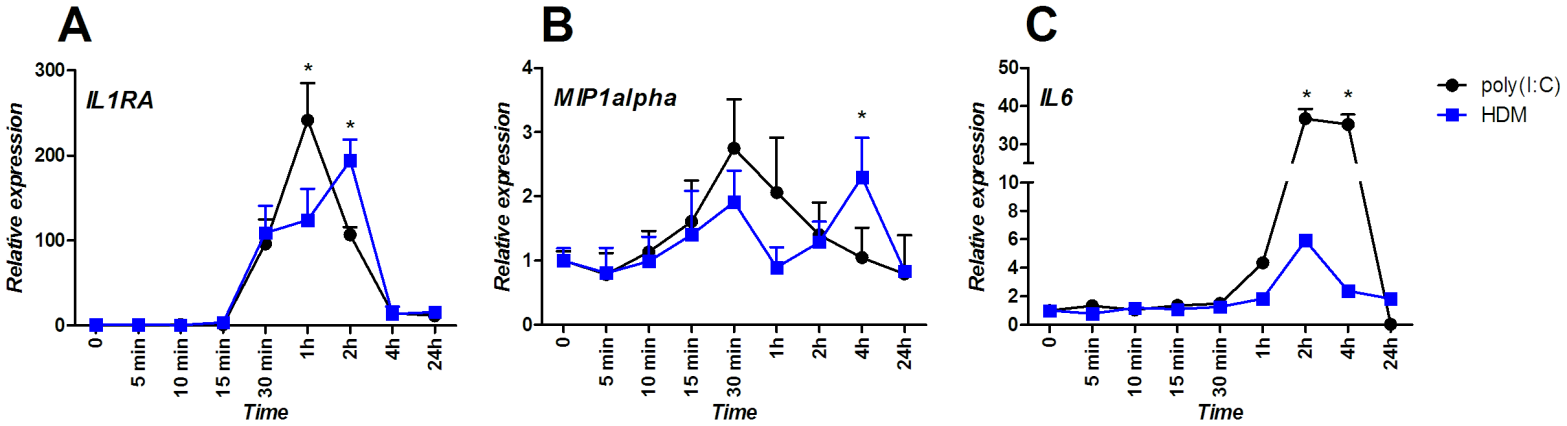
Detailed gene expression analysis. Temporal patterns of genes: A) and B): differentially expressed after stimulation with HDM or poly(I:C); C) with similar expression pattern triggered by HDM or poly(I:C). Each time point represents an average value of three biological replicates ± standard deviation. Statistically significant differences (p<0.05) in the gene up-regulation levels between HDM and poly(I:C) stimulation are indicated (*).

Quantitative PCR analysis indicated a clear overlap in gene expression in response to HDM and poly(I:C) stimulation, which led us to hypothesize that this overlap would be mirrored in mediators production by these cells. We therefore measured baselines and induced levels of 30 cytokines, chemokines, and growth factors after HDM or poly(I:C) exposure. As can be seen in table 1, the responses to 24 hours stimulation by HDM and poly(I:C) are rather similar, albeit with some quantitative differences. In the 24 hours supernatants, for four mediators (IL1-RA, IL-17, IFN-α, and MIP1-α) we could detect expression at baseline and with identical levels of induction by HDM and poly(I:C). To a certain extent this similarity also applied to FGF-basic protein where both HDM and poly(I:C) failed to induce baseline levels and to 15 other mediators that could not be detected at baseline or after HDM and poly(I:C) exposure. Quantitative differences could be detected in six mediators affected by both stimuli, but induced to a higher level after poly(I:C) exposure (IL2-R, IL-6, IL-8, MCP-1, and MIG) or after HDM exposure (HGF). Interestingly, only four mediators (IL-12, IP-10, RANTES, and VEGF) where up-regulated specifically by poly(I:C) and not by HDM. In contrast to protein data obtained from the 24 hour cell-free supernatants, 8 hours exposure to HDM resulted in a statistically significant up-regulation of IL-6 and IL-8 only. Stimulation with the TLR-3 agonist led to a significant induction of the following mediators: IL1-RA, MCP-1, MIP1-α, IP-10, RANTES, and VEGF. For all measured mediators in the 8 hour supernatant, fold changes of their production and secretion are somewhat higher, probably due to reduced baseline production. Induction of IL-6 and IL-8 is still significantly higher for the viral analogue and IP-10, RANTES, and VEGF were up-regulated specifically by poly(I:C) and not by HDM, which is in line with the 24 hours data. However, production patterns of other mediators such us IL-1-RA and MIP1-α do not longer resemble that of the 24 hour time point, where no statistically significant differences between the two stimuli were observed. 8-hour stimulation with poly(I:C) significantly affected these two mediators release, whereas HDM did not. To verify whether production of these mediators is controlled at mRNA level, we examined the expression profiles of the *IL1RA* and *MIP1alpha* genes over a 24-hour time course in response to HDM and poly(I:C) triggering. Both genes were up-regulated by poly(I:C) or HDM to similar levels, however the allergen induced their maximal expression somewhat later. The maximum expression of *IL1RA* was observed 1 hour after poly(I:C) stimulation, whereas for HDM the maximum level of *IL1RA* was an hour later (Figure 4A). The *MIP1alpha* gene reached its maximal expression level already 30 minutes after poly(I:C), in contrast to four hours for HDM (Figure 4B). Additionally, we compared expression profiles of *IL6* in response to both triggers, as IL-6 release was observed both 8 and 24 hours after stimulation. As expected, the *IL6* gene was up-regulated by both triggers in a similar time frame and its maximal expression level is reached two hours after cell exposure to HDM or poly(I:C) (Figure 4C). mRNA up-regulation levels of *IL6* to some degree resemble the differences in secreted protein concentration.

**Table 1:** Mediators secreted 8 or 24(I:C) or HDM.

Mediator	HBSS		HDM		poly(I:C)		FC HDM		FC poly(I:C)	
	8h	24h	8h	24h	8h	24h	8h	24h	8h	24h
*Cytokines up-regulated by HDM and poly(I:C)*										
**IL1-RA**	16.7	31.6 (±4.7)	16.7	230.0 (±18.6)	242.8 (±29.0)	260.9 (±78.0)	1.0	7.3 (±1.2)^#^	14.5 (±1.8)^#^*	8.3 (±2.7)^#^
**IL-2R**	b.d.	52.6 (±26.0)	b.d.	104.2 (±27.5)	b.d.	410.5 (±35.6)	x	2.0 (±1.1)^#^	x	7.8 (±3.9)^#^*
**IL-6**	2.6	20.0 (±10.0)	12.6 (±2.7)	50.0 (±3.1)	453.8 (±159.0)	636.9 (±45.7)	4.84 (±0.9)	2.2 (±1.2)^#^	174.6 (±61.0)^#^*	31.8 (±10.7)^#^*
**IL-8**	5.8	260.7 (±37.0)	104.7 (±9.0)	1088.0 (±42.6)	948.0 (±194.0)	4471.6 (±300.2)	18.0 (±3.7)	4.2(±0.6)^#^	163.4 (±33.0)^#^*	17.1 (±2.7)^#^*
**IL-17**	b.d.	5.3	b.d.	28.0 (±12.0)	b.d.	23.2 (±15.8)	x	5.2 (±2.2)^#^	x	4.3 (±2.9)^#^
**IFN-α**	b.d.	16.6 (±7.9)	b.d.	40.8 (±3.8)	b.d.	44.5 (±8.7)	x	2.5 (±1.2)^#^	x	2.7 (±1.4)^#^
**MCP-1**	19.1	192.3 (±18.4)	19.1	437.8 (±24.4)	137.9 (±35.2)	801.9 (±39.3)	1	2.3 (±0.2)^#^	7.2 (±2)^#^*	4.2 (±0.4)^#^
**MIP1-α**	3.4	17.5 (±11.1)	3.4	60.2 (±7.3)	17.7 (±3.4)	63.2 (±13.3)	1	3.4 (±2.2)^#^	5.3 (±1.0)^#^*	3.6 (±2.4)^#^
**MIG**	b.d.	5.7 (±0.7)	b.d.	15.4 (±0.6)	b.d.	28.4 (±2.2)	x	2.7 (±0.4)^#^	x	5 (±0.7)^#^
**HGF**	b.d.	3.2	b.d.	45.5 (±4.9)	b.d.	15.3 (±8.9)	x	14.0 (±1.5)^#^	x	4.7 (±2.8)^#^*
*Cytokines up-regulated by poly(I:C)*										
**IP-10**	1.9	1.9	1.9	1.9	18.4 (±3.1)	319.6 (±15.4)	1	1	9.68 (±2.1)^#^*	163.0 (±7.9)^#^*
**RANTES**	5.4	48.2 (±13.7)	5.4	32.4 (±8.0)	233.1 (±70.5)	260.1 (±17.1)	1	0.7 (±0.21)	43.2 (±13.0)^#^*	5.4 (±1.6)^#^*
**IL-12**	b.d.	9.3	b.d.	9.3	b.d.	33.0 (±0.8)	x	1	x	3.6 (± 0.1)^#^*
**VEGF**	3.5	105.4 (±16.0)	3.5	153.2 (±17.2)	39.7 (±8.3)	264.1 (±12.1)	1	1.4 (±0.9)	11.3 (2.4)^#^*	2.5 (±1.5)^#^*
*Cytokines not affected by either stimuli*										
**FGF-basic**	b.d.	29.2 (±3.4)	b.d.	29.8 (±4.3)	b.d.	43.0 (±7.7)	x	1.0 (0.1)	x	1.5(±0.3)

Cell-free supernatants were analyzed for the presence of cytokines and chemokines. The first part of the table shows mediators induced by 8 or 24 hours stimulation with both HDM and poly(I:C). In the second part, mediators induced only by poly(I:C) was collected. The bottom of the table shows a cytokine which was not affected by either of the stimuli. The concentration values are given in pg/mL and standard deviations are in brackets and represent the average of three biological replicates. Fold changes (FC) were calculated as a ratio between stimulated and control cells and if significant (p<0.05), marked with (^#^). Significant differences (p<0.05) between levels of induction by HDM and poly(I:C) are marked with (*). B.d – below the detection limit.

### Pre-exposure to HDM affects cells responses to poly(I:C) stimulation

The NCI-H292 cell line produces and secretes a variety of inflammation-related cytokines/chemokines in response to HDM or the synthetic dsRNA stimulation. The evidence of *TLR3* down-regulation by HDM provocation in airway epithelium led us to investigate the cytokine production profile by cells pre-exposed for 24 hours to HDM and then subsequently stimulated with poly(I:C) for additional 8 or 24 hours. Prior to poly(I:C) stimulation, the allergen was removed from the cell culture medium. The virus in an allergy mimic setting revealed an enhanced production of IL-8 (Table 2) when compared to the cytokines production levels from cells pre-exposed to HBSS and then followed by 8/24-hour stimulation with poly(I:C). Interestingly, in response to poly(I:C), release of three out of four cytokines that were exclusively affected by the TLR-3 agonist exposure (Table 1), namely IL-12, RANTES, and IP-10 – was significantly reduced when pre-exposed to HDM. Production of IL-6 and MIP1-α was down-regulated by the HDM pre-exposure as well. No statistically significant changes in the cytokine levels were observed for the following cytokines: IL1-RA, VEGF, FGF-basic, and MCP-1.

**Table 2:** Mediators secreted 8h or 24h after poly(I:C) stimulation alone or additionally pre-exposed for 24 hours to HDM.

Mediator	24h HBSS + xh poly(I:C)		24h HDM + xh poly(I:C)		FC	
	8h	24h	8h	24h	8h	24h
*Up-regulated cytokines*						
**IL-8**	514.0 (48.0)^#^	8146.0 (±568.0)^#^	544.0 (±52.0)^#^	12135.0 (570.0)^#^	1.0 (±0.1)	1.5 (±0.1)*
*Down-regulated cytokines*						
**RANTES**	79.0 (±2.0)^ #^	565.0 (±48.0)^#^	45.0 (±13.0)^ #^	305.0 (±14.0)^#^	0.6 (±0.2)*	0.5 (±0.1)*
**IP-10**	579.0 (±87.0)^#^	6334.0 (±336.0)^#^	346.0 (±55.0)^#^	5129.0 (±564.0)^#^	0.60 (±0.1)*	0.8 (±0.1)*
**IL-12**	b.d.	40.0 (±2.0)^#^	b.d.	30.0 (±3.0)	x	0.7 (±0.1)*
**IL-6**	214.0 (±22.0)^#^	7709.0 (±1224.0)^#^	187.0 (±16.0)^#^	3802.0 (±301.0)^#^	0.9 (±0.1)	0.5 (±0.1)*
**MIP1-α**	50.0 (±4.0)^#^	51.0 (±2.0)^#^	22.0 (±0.1)	25.0 (±2.1)	0.4 (±0.1)*	0.5 (±0.0)*
*Not affected cytokines*						
**VEGF**	89.0 (±10.1)^#^	640.1 (±65.0)^#^	109.1 (±30.2)^#^	705.2 (±77.3)^#^	1.2 (±0.4)	1.1 (±0.2)
**IL1-RA**	552.0 (±36.2)^#^	1332.2 (±150.1)^#^	576.0 (±78.1)^#^	1431.2 (±223.0)^#^	1.0 (±0.2)	1.1 (±0.2)
**MCP-1**	233.0 (±24.0)^#^	359.2 (±24.2)^#^	244.4 (±20.1)^#^	373.0 (±16.2)^#^	1.0 (±0.1)	1.0 (±0.1)
**FGF-basic**	b.d.	13.0 (±1.0)	b.d.	14.1 (±2.0)	b.d.	1.1 (±0.1)

Cell-free supernatants were analyzed for the presence of cytokines and chemokines in relation to stimulation by poly(I:C) with or without HDM pre-exposure. The table shows cytokines that are: HDM-enhanced in response to poly(I:C); HDM-down-regulated in response to poly(I:C) and not affected by HDM pre-exposure. The concentration values are given in pg/mL and standard deviations are in brackets and represent an average of a triplicate experiment. Fold changes (FC) were calculated as a ratio between HDM pre-stimulation followed by poly(I:C) exposure condition to HBSS pre-exposure followed by poly(I:C) stimulation condition. Fold changes and values were considered significant if p<0.05. Statistically significant induction of cytokines production by poly(I:C) compared to non-induced control conditions is marked with (^#^) and significant effect of HDM pre-exposure to the FC values are marked with (*). B.d – below the detection limit.

## Discussion

In this manuscript we have shown that the temporal expression pattern of the genes that we had previously defined as the common response to house dust mite allergen in nose and lung epithelial cells [9], [10] to a large degree overlaps with that of the anti-viral response. However, a small number of the transcription factors have a temporal pattern that is specific for each stimulus. This data provides the first insight into the molecular mechanism that could be responsible for the many clinical links that exist between allergy and viral infections. Inflammatory molecular responses are often attributed to the induction of NF-κB and AP-1, however it is also clear that they are not the only transcription factors involved [9], [16], [17].

We have previously shown [9], [10] that expression profiles of multiple genes from the NF-κB and AP-1 transcription factor families (*NFKB1*, *NFKB2*, *FOS, JUN*, *JUNB,* and *FOSL1*) as well as unrelated factors like *MYC*, *ATF3*, *EGR1,* and *DUSP1* define the differences between the response in healthy and allergic individuals (Figure 1). A direct comparison of all the responses in healthy, allergic epithelium and the H292 cell line revealed a core-response to house dust mite and this response may potentially contribute to the “steady state” induced after prolonged exposure to allergen as the micro-array data was collected 24 hours after the exposure [10]. To investigate this core response in greater detail we established the temporal aspect of this response.

The temporal response in airway epithelial cells we observed after HDM exposure can largely be described as a two-phase response with some transcription factors induced rather quickly after HDM allergen challenge and others relatively late after exposure. The responses of *EGR1*, *ATF3*, *DUSP1*, and *FOS* are already up-regulated after 30 minutes, reach their peak of expression at 60 minutes and are back at baseline levels after 120 minutes. Most of the other transcription factors increase their expression after 60 minutes and do not reach their maximal levels before 120 (*JUN*) or 240 minutes (*MYC*, *FOSL1*).

It is interesting to note that this dichotomy of responses is reflected by the expression pattern observed for these transcription factors in primary healthy and allergic epithelial cells. In primary epithelial cells some transcription factors have a low expression pattern at baseline and fail to be up-regulated in allergic individuals upon 24 hours stimulation with house dust mite allergen, while they are up-regulated in healthy individuals. *ATF3*, *EGR1*, *DUSP1*, and *FOS*, which all show this difference in expression pattern in allergic and healthy individuals, do all belong to the early induced class of genes described in this manuscript. In contrast, the late induced transcription factors *MYC* and *FOSL1* both are in a constantly activated state in primary epithelial cells from allergic individuals, meaning that at baseline they already display a high level of expression which remains unchanged after *in vitro* house dust mite exposure [9].

Some of the early-regulated genes have been previously reported to down-regulate induced or ongoing inflammatory responses. Mostecki and colleagues showed that expression of *EGR1* is able to down-regulate both basal and LPS induced responses in mouse macrophages [18] and another report demonstrated that the interplay between EGR-1 and AP-1 is important for modulation of stress-triggered responses [8]. *ATF3* has been linked with the inhibition of IL-6 and IL-12B production [18], [19]. A homodimeric ATF-3 protein was also reported to repress the expression of a wide spectrum of pro-inflammatory cytokines [20] and compete with promoter binding sites of NF-κB regulated genes [19]. The timing of RNA expression of *ATF3* and *EGR1* would be in line with this potential role of an inflammation modulator as early RNA expression would allow sufficient time for translation, so that they would be able to down-regulate the potentially following NF-κB and AP-1 responses [21]. Therefore, the role of the early response genes *ATF3* and *EGR1* might well be to shut down the pro-inflammatory response induced by NF-κB and AP-1 transcription factor family members [22].

Another important observation comes from the direct comparison of HDM and TLR-3-agonist induced responses. The temporal expression profiles for most of the transcription factors we have evaluated are similar after both stimuli, which may represent a first step towards a possible explanation of how viral infections and responses to allergens may affect each other. Despite this high degree of overlap of both responses, there is also some specificity. For *ATF3* and *FOS* the response after poly(I:C) stimulation is delayed and reduced in comparison to the HDM response, while *NFBK1* is strongly induced by poly(I:C) and not by HDM.

The commonalities of the HDM and poly(I:C) response on the transcription factor level are also reflected at the mediator level. Most of the tested mediators have similar induction patterns for HDM and poly(I:C). One mediator is not induced at all by either of stimuli, five mediators have identical levels of induction, while five mediators are differentially induced by both stimuli. Only four mediators are induced specifically by one stimulus (IL-12, IP-10, VEGF, and RANTES), in this case poly(I:C). This high degree of overlap could potentially explain part of the mechanism by which virus induces allergic exacerbations and is also probably the reason that in state-of-the-art reviews on the impact of viral infections on allergic exacerbations it was not possible to find any specific differences given that research in this field has been limited to only restricted number of mediators [23], [24]. Whether the commonalities and differences at the mediator level is a direct reflection of the commonalities and differences at the transcription factor level remains to be explored.

Interestingly, our data reveal that there is some specificity that only emerged by screening multiple mediators. Given the still limited number of entities tested and their inflammatory nature this approach can hardly be called unbiased, but does show the benefits of such an approach. The specific anti-viral response induced cytokines included IFN-inducible genes such as IP-10 and RANTES accompanied by IL-12 and this production profile may reflect the specific needs of this response. Induction of IL-12 can facilitate the anti-viral Th1-response, although in our system the source is airway epithelial cells rather than archetypical dendritic cells. Given the mutual inhibitory actions of Th2 on Th1 responses, this epithelial IL-12 response may allow or help the anti-viral response even in an allergic background. Recruitment and activation of neutrophils, macrophages, and Th1 cells mediated by viral TLR-3 ligation combined with IL-8 and RANTES may imply an important role of viral exacerbations of allergic asthma and COPD [25]. Additionally, RANTES produced during severe respiratory syncytial virus infection may have a significant impact on the inflammatory response during subsequent allergic challenge reflected in an increase of a number of peribronchial eosinophils [26].

While comparing mediator levels in supernatants of 8 and 24-hour stimulated cells, some discrepancies emerged. Detectable levels of only a few cytokines produced by the H292 cell line could be measured 8 hours after exposure to poly(I:C). Only two mediators, namely IL-6 and IL-8, were affected by HDM stimulation. Perhaps it is not that surprising, as some of the chemokines are not produced abundantly by the cell line and therefore 8 hours exposure would simply be too short to accumulate measurable amounts of protein. Nevertheless, release level of IL1-RA and MIP1-α in response to HDM and poly(I:C) is no longer similar as measured in the 24-hour supernatant, suggesting a differential regulation of their production by the two triggers already at the mRNA level. *IL1RA* and *MIP1alpha* gene expression levels revealed a delayed cell response to HDM stimulation if compared to that of poly(I:C), perhaps explaining the absence of IL1-RA and MIP1-α in the 8-hour supernatant. Moreover, one of two cytokines that we are able to measure already 8 hours after cell exposure to HDM, namely IL-6, reveals a similar timing of induction at the mRNA level by both triggers. Therefore, observed expression profiles suggest that regulation of IL1-RA, MIP1-α, and IL-6 production takes place, at least partially, at the mRNA level.

It has been observed that viral clearance in allergic individuals takes longer when compared to non-atopic patients and virus infection symptoms may be more severe if accompanied by allergy [13]. To mimic the presence of a virus in an allergy setting we pre-exposed cells to HDM and then stimulated with poly(I:C). Production and release of one pro-inflammatory mediator (IL-8) in response to poly(I:C) was significantly enhanced by HDM pre-stimulation and release of five other cytokines, namely IP-10, IL-12, RANTES, IL-6, and MIP1-α was dramatically reduced by HDM pre-exposure. Additionally, production of four cytokines was not affected by the allergen pre-stimulation. Interestingly, release of three out of four cytokines, which are specifically induced by 24 hours poly(I:C) stimulation was now reduced by the allergen pre-incubation. Given that airway epithelium exposure to HDM has been linked with the *TLR-3* gene expression down-regulation [9], it does not come as a surprise that the production of some anti-viral specific mediators becomes impaired in an allergy setting. As RANTES, IP-10, and IL-6 have been associated with rhinoviruses clearance [27]-[29], their reduced production may perhaps indicate why some atopic individuals suffer from a pro-longed rhinoviral infection.

The H292 cell line was used in this study as a model of human airway epithelium interactions with allergens and viral dsRNA. We have previously performed an extensive analysis of similarities between the responses of H292 and primary epithelium to HDM, and even though we concluded that H292 cannot be used as a *detailed* model of the responses of primary nasal epithelium to HDM, it does help to understand the contribution of airway epithelium to the basic responses to an allergen [10]. Given that *in vitro* responses to HDM of epithelial cells isolated from non-allergic donors vary significantly between the different individuals (data not shown), using a cell line as a model may help to understand how upper and/or lower airway contribute to allergen-driven immune responses without the confusion of the intra-individual variances.

To our knowledge, no data is available on similarities between responses of H292 and primary epithelial cells to poly(I:C). We are currently extending the study with primary nasal epithelial cells from non-asthmatic and non-allergic individuals exposed to poly(I:C) and preliminary data suggest that expression profiles of the majority of transcription factors and pro-inflammatory cytokines investigated in this study are quite similar between H292 and primary airway epithelial cells.

Our data provides a first insight into both the commonalities and differences of the responses to an allergen and to a virus. If we were allowed to generalize our observations we could speculate the specificity of both allergic and anti-viral responses would be down-stream of *ATF3*, *NFKB1*, and *FOS*. In some sense this would be similar to the fate of T helper cells where single transcription factors are held responsible for the specificity of the response [30] with T-bet responsible for the Th1 response, GATA-3 responsible for the Th2, RORC for the Th17, and Fox-p3 for the regulatory response.

The high degree of overlap between the HDM allergen and responses to viral infection is intriguing. As our HDM extract lacks any biological relevant LPS, the overlap of both responses is not a trivial consequence of a similarity in TLR-4 and TLR-3 responses and would suggest that the responses to the HDM allergen and a poly(I:C) share similar requirements. This is a remarkable notion as in the case of a viral infection one can easily imagine that the immune system needs to be activated to fight off a potential threat, whereas after exposure to a generally considered harmless allergen one would not expect that activation of the immune system would be required. However, a few points need to be considered. Firstly, even if an allergen could be seen as harmless, the immune system would still need to make an informed decision. Triggering a response to an allergen is therefore required, although this response does not necessarily need to be the same as the response triggered by a virus. Secondly, allergens are perhaps not as innocent as would be inclined to think, as through their protease activity they may disturb the barrier function of the epithelium, so that the immune response triggered by allergens could be seen as an advanced warning signal. Finally, viruses may try to evade a strong immune response against them by triggering a Th2-response that might attenuate the Th1 response which would normally be required for an efficient eradication of the virus [31].

Although the choice of the genes investigated in this study was based on solid evidence for their importance in airway epithelium responses to HDM, it is clear that other environmental factors, such as diesel fume, cigarette smoke or scratch injury may also affect their expression. Among others, EGR-1 has been associated with cigarette smoke induced COPD [32], smoking has also been associated in ATF-3 and c-JUN induction in human lung cells [33], [34] and diesel fume particles have been involved in up-regulation of *FOS* expression in human bronchial epithelium [35]. A well-known association of these environmental factors with asthma/allergy development risk may perhaps explain the similarities of the ‘shared response’ gene induction pattern between an allergen and some allergy/asthma causative factors.

The regulatory mechanisms activated by HDM and poly(I:C) exposure are closely linked with the production of functional and biologically active mediators. Majority of the genes from the ‘shared response’ of airway epithelium to HDM consisted of transcription factors with known affinity to certain DNA motifs in promoter regions of some cytokines/chemokines. For instance, NF-κB protein is known to regulate the expression of all mediators investigated in this study. Binding sites of c-JUN or c-FOS have been displayed in the promoter regions of e.g. *IL6* and *IL8*. Additionally, binding sites of the early-response transcription factors are found in the promoter sites of the late-phase genes and some of the genes possess the function of their own auto-regulation eventually influencing the expression and production level of targeted mediators. Understanding the mechanisms behind the cellular responses to HDM and poly(I:C) may enable interpreting for instance why pre-exposure to an allergen leads to the deregulation of anti-viral responses and therefore may help prevent the clinical consequences of the interplay of the two triggers.

The analysis of the “HDM core response” genes [10] in response to HDM in the airway epithelium revealed a dysregulation of genes directly linked to the defense against viral infections, namely *TLR3, TICAM1,* and *IVNS1ABP*. Another study has shown a significantly down-regulated baseline expression of the *TLR3* gene in nasal epithelium of house dust mite allergic individuals [9]. Here we show that not only are the airway epithelium responses to HDM or a viral trigger similar, but also that they are linked. *In vitro* pre-exposure to HDM led to a significant deregulation of the poly(I:C) responses, what may result in direct or indirect clinical consequences if the same observations are made *in vivo*.

Generation of a particular micro-environment created by allergen-activated epithelial cells in healthy individuals may directly or indirectly contribute to the maintenance or the development of local homeostasis that may be important for sustaining the balance between allergen-specific Treg1 cells and Th2 cells which is claimed to be decisive in the development of allergy or for dampening any inappropriate local immune responses [36].
